# PCR detection of *Toxoplasma gondii* DNA in fecal samples from stray cats in Bangkok Metropolitan, Thailand

**DOI:** 10.1016/j.fawpar.2026.e00339

**Published:** 2026-04-25

**Authors:** Chanya Kengradomkij, Wissanuwat Chimnoi, Ketsarin Kamyingkird, Adrian B. Hehl, Tawin Inpankaew

**Affiliations:** a Department of Parasitology, Faculty of Veterinary Medicine, Kasetsart University, Bangkok 10900, Thailand; b Laboratory of Molecular Parasitology, Institute of Parasitology, Vetsuisse and Medical Faculty, University of Zurich, CH-8057 Zurich, Switzerland; c One Health Institute, Vetsuisse, Science, and Medical Faculties, University of Zurich, CH-8057 Zurich, Switzerland

**Keywords:** *Toxoplasma gondii*, PCR detection, Environmental contamination, Temple cats, Public health risk, Fecal samples

## Abstract

*Toxoplasma gondii* is a globally important zoonotic parasite, with domestic cats serving as the definitive host by shedding oocysts into the environment, where they undergo sporulation, a process that increases the resistance of the oocyst wall and facilitates long-term environmental persistence. In densely populated urban environments, free-roaming cat populations may contribute to environmental contamination and pose a potential public health risk. Here, we assessed the presence of *T. gondii* DNA in fecal samples from stray cats residing in temple communities across Bangkok, Thailand, using molecular detection.

Fecal samples collected from stray cats across multiple districts of Bangkok were screened by PCR. *T. gondii* DNA was detected at low prevalence but across a broad geographic range, suggesting widespread environmental exposure. No clear associations were observed between PCR detection of *T. gondii* DNA and assessed animal-related or management-related factors.

These findings provide molecular evidence of the presence of *T. gondii* in Bangkok temple environments. Although PCR-based detection does not confirm active oocyst shedding, the widespread distribution of positive samples underscores the need for continued environmental surveillance and integrated One Health approaches to mitigate human and animal exposure to toxoplasmosis in urban settings.

## Highlights

• Molecular detection of *Toxoplasma gondii* DNA in stray cats in Bangkok.
• Low prevalence but wide geographic distribution observed.
• No significant association with host or management factors.
• Evidence suggests environmental contamination in temple settings.
• Supports the need for One Health surveillance strategies.

## 1 Introduction

*Toxoplasma gondii* is an obligate intracellular coccidian parasite that infects a wide range of warm-blooded animals, including humans (Su et al., 2022). Toxoplasmosis is a globally important zoonotic infection. Infection in immunocompetent individuals is typically asymptomatic or mild, whereas severe disease mainly occurs in immunocompromised patients and in cases of congenital infection (Montoya and Liesenfeld, 2004; Djurković-Djaković et al., 2019). In humans, infection may result in lymphadenitis, encephalitis, and chorioretinitis (Weiss and Dubey, 2009), and it poses a particular risk during pregnancy due to the potential for congenital transmission and severe fetal outcomes (Montoya and Liesenfeld, 2004; Chaudhry et al., 2014). Humans acquire infection primarily through the consumption of raw or undercooked meat containing tissue cysts or through ingestion of food or water contaminated with oocysts shed in cat feces (Jung et al., 2015).

Domestic cats and other felids serve as the definitive hosts of *T. gondii*, as sexual reproduction of the parasite occurs exclusively in their intestinal epithelium, leading to the shedding of oocysts into the environment (Elmore et al., 2010; Shapiro et al., 2019). After being shed into the environment, oocysts undergo sporulation and become infective, and their multilayered wall enables them to persist in soil and water for extended periods, in some cases for more than a year (Al-Malki, 2021; Shapiro et al., 2019; Elmore et al., 2010). As a result, environmental contamination with oocysts represents an important source of infection for intermediate hosts, including humans and livestock (Elmore et al., 2010; Robert-Gangneux and Dardé, 2012; Stelzer et al., 2019).

In Thailand, stray cats are commonly found in temple compounds, where they are frequently fed and cared for by monks, nuns, and local caretakers. Temples provide relatively stable shelter and food resources, often resulting in high-density stray cat populations. The city of Bangkok contains nearly 500 temples (National Office of Buddhism, 2024), many of which host substantial numbers of free-roaming cats. These temple grounds serve not only as religious sites but also as community gathering spaces, increasing the likelihood of direct or indirect human contact with cats and contaminated environments. Although systematic census data are limited, the widespread presence of stray cats in temple settings suggests that contamination of food, water, and soil with oocysts from cat feces may represent a relevant public health concern in urban Bangkok.

Diagnosis of *T. gondii* infection in cats can be approached through microscopic detection of oocysts in feces, bioassays, molecular methods, or serological detection of antibodies. Importantly, cats typically shed oocysts for a short period only following primary infection, during which large numbers of oocysts are excreted (Dubey, 1995; Dubey, 2022). After the patency period no parasites remain in the intestine and cats do not shed oocysts anymore after subsequent infections with tissue cysts in prey animals (Ramakrishnan et al., 2019). Clinical signs during the acute phase of the primary infection are usually absent, mild, or non-specific (Elmore et al., 2010; Jokelainen et al., 2012), rendering clinical examination unreliable for identifying cats that are shedding oocysts. Consequently, diagnosis of acute toxoplasmosis based on clinical signs is not informative, and identification of cats in the process of oocyst shedding is inherently challenging due to the short temporal window.

Serological testing is therefore commonly used in epidemiological studies, as antibodies persist long after infection (Dubey, 1995; Dabritz and Conrad, 2010). However, seropositive cats are generally considered to pose a limited immediate risk for environmental contamination, since oocyst shedding typically precedes seroconversion (Lappin, 2010). In contrast, detection of *T. gondii* DNA in fecal samples strongly indicates the presence of the parasite, although it does not definitively confirm active oocyst shedding, and can be useful for assessing potential environmental contamination and public health risk.

Microscopic examination of fecal samples has limited sensitivity and cannot reliably distinguish *T. gondii* oocysts from those of other coccidian parasites with similar morphology (Bawm et al., 2020; Dubey et al., 2020; Igreja et al., 2021; Sheng et al., 2023). Bioassays, although sensitive, are costly, time-consuming, and associated with ethical and biosafety concerns (Karakavuk et al., 2022; Veronesi et al., 2017). Molecular techniques, particularly PCR-based methods, offer high sensitivity and specificity and allow accurate differentiation of *T. gondii* from related coccidia, making them especially suitable for detecting parasite DNA in feline fecal samples (Igreja et al., 2021; Liu et al., 2015).

In Thailand, most studies on *T. gondii* in cats have focused on seroprevalence. In Bangkok, reported seroprevalence in pet cats ranges from 1.5% to 10.1% (Inpankaew et al., 2021; Sukhumavasi et al., 2012; Sukthana et al., 2003), while higher rates have been observed in stray cats, ranging from 4.8% to 11.5% (Jittapalapong et al., 2007; Jittapalapong et al., 2010; Inpankaew et al., 2021; Kengradomkij et al., 2018). A study from western Thailand reported a seroprevalence of 8.3% in farm cats (Arunvipas et al., 2013). In contrast, molecular detection of *T. gondii* oocysts in cat feces has been reported only from northern (Suwancharoen et al., 2022) and southern Thailand (Chemoh et al., 2016), and data from Bangkok remain limited.

The objectives of the present study were to detect *T. gondii* DNA in fecal samples from stray cats residing in temple communities across Bangkok using PCR, and to assess potential risk factors associated with parasite detection. By focusing on molecular detection, this study aims to provide insight into potential environmental contamination and the potential public health relevance of stray cat populations in an urban temple setting.

## 2 Materials and methods

### 2.1 Study area and animal samples

Fecal samples were collected from stray cats residing in temple compounds across 26 districts of Bangkok, Thailand, representing areas with varying human population densities. Individual samples were obtained by rectal swabbing performed by trained technicians or veterinarians. Each swab was placed in a clean container and stored at 4 °C until further processing.

Cats of both sexes and a range of estimated ages were included. Between 5 and 15 cats were sampled per temple using convenience sampling. Data on animal-related and management-related factors, including sex, age, feeding practices, roaming status, housing conditions, veterinary care, and deworming history, was collected using questionnaires completed by monks, nuns, or animal caretakers responsible for the cats.

All procedures were approved by the Animal Ethics Committee of the Faculty of Veterinary Medicine, Kasetsart University, and were conducted in accordance with the Ethics of Animal Experimentation of the National Research Council of Thailand (approval number ACKU60-VET-007).

### 2.2 Molecular detection of *T. gondii* using PCR

#### 2.2.1 DNA extraction

After collection, cotton swabs containing fecal material were stored at 4 °C until DNA extraction. Prior to extraction, each swab was immersed in normal saline and vortexed to release all fecal material into the solution, which was then used in its entirety for DNA extraction. Genomic DNA was extracted using a commercial stool DNA extraction kit (NucleoSpin® DNA Stool, MACHEREY-NAGEL, Germany), following the manufacturer's instructions with minor modifications. The final elution volume was reduced to 100 μL to increase DNA concentration. Extracted DNA was stored at −20 °C until PCR analysis.

#### 2.2.2 Conventional PCR and DNA sequencing

Detection of *T. gondii* DNA was performed by conventional PCR using primers TOX4 (5’-CGCTGCAGGGAGGAAGACGAAAGTTG-3′) and TOX5 (5’-CGCTGCAGACACAGTGCATCTGGATT-3′), which amplify a 529 bp non-coding repetitive DNA fragment specific to *T. gondii* (Homan et al., 2000). Each PCR reaction was performed in a final volume of 20 μL containing PCR buffer, MgCl₂, dNTPs, primers, Taq DNA polymerase (Invitrogen™, Thermo Fisher Scientific, USA), and 2 μL of template DNA.

DNA extracted from *T. gondii* tachyzoites was used as a positive control, while distilled water served as a negative control. Amplification was carried out under the following conditions: initial denaturation at 94 °C for 7 min, followed by 35 cycles of denaturation at 95 °C for 1 min, annealing at 60 °C for 1 min, and extension at 72 °C for 1 min, with a final extension at 72 °C for 10 min using a thermal cycler (T100 thermal cycler, Bio-Rad). PCR products were separated by electrophoresis on a 1.5% agarose gel and visualized using a Gel Doc XR+ system (Bio-Rad). Samples showing a band of approximately 500–550 bp were considered positive.

Selected PCR-positive products were excised from the gel and purified using a gel extraction kit (NucleoSpin® Gel and PCR Clean-up, MACHEREY-NAGEL GmbH & Co., Germany). Purified amplicons were subjected to Sanger sequencing (Macrogen, Seoul, Republic of Korea). Obtained sequences were analyzed using the BLAST algorithm against the NCBI GenBank database to confirm species identity.

### 2.3 Statistical and risk factor analysis

The prevalence of *T. gondii* DNA detection was calculated as the proportion of PCR-positive samples among the total number of cats examined. Associations between PCR positivity and categorical variables, including sex, age group, roaming status, feeding practices, veterinary care, and deworming status, were evaluated using the chi-square (χ^2^) test. Statistical analyses were performed using Number Cruncher Statistical System (NCSS) version 2000 (Kaysville, Utah, USA). A *p*-value of less than 0.05 was considered statistically significant.

## 3 Results

### 3.1 Study population

A total of 652 fecal samples were collected from stray cats residing in temple compounds across 26 districts of Bangkok. Of these, 272 cats (41.7%) were male and 380 (58.3%) were female. Based on estimated age, 459 cats (70.4%) were ≤ 3 years old and 193 cats (29.6%) were older than 3 years. The mean estimated age of cats included in the study was 2.81 ± 1.86 years.

### 3.2 Detection of *T. gondii* DNA in fecal samples

**Fig. 1:**
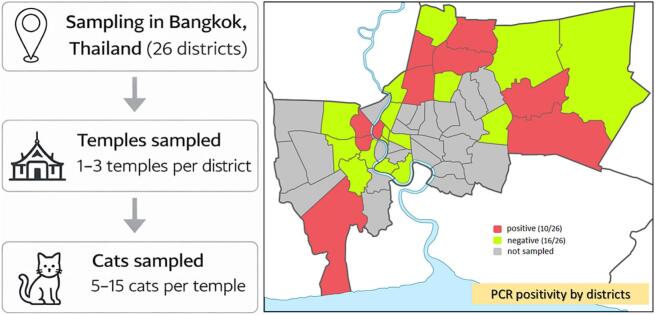
Study workflow and geographic distribution of *Toxoplasma gondii* PCR positivity in temple cats in Bangkok. Sampling design across 26 Bangkok districts (colored map areas), with 1–3 temples per district and approximately 5–15 cats sampled per temple. Fecal samples (rectal swabs) were collected for molecular detection of *T. gondii* DNA. The geographic distribution of PCR positivity by district is indicated, with positive districts shown in red (10/26), negative districts in green (16/26), and unsampled districts in gray. (For interpretation of the references to colour in this figure legend, the reader is referred to the web version of this article.)

*T. gondii* DNA was detected in 16 of the 652 fecal samples, corresponding to an overall molecular prevalence of 2.5% (95% CI = 1.41–3.95%). PCR-positive samples were identified in 38.5% (10/26) of the surveyed districts (95% CI = 20.2–59.4%) (Fig. 1), indicating that the parasite was geographically widespread despite the low overall prevalence.

The prevalence varied markedly between districts, ranging from 0.0% to 20.0%. The highest proportion of PCR-positive samples was observed in Bangkok Yai District (3/15; 20.0%), followed by Min Buri (3/35; 8.6%), Lat Krabang (2/32; 6.3%), and Lak Si (2/34; 5.9%). No PCR-positive samples were detected in 16 districts (Table 1).

**Table 1:** Detection of *Toxoplasma gondii* DNA from stray cats in 26 districts of Bangkok, Thailand.

District	No. of PCR positive/No. of samples	Percentage (%)	95% CI (%)
Bangkok Yai	3/15	20.0	4.33–48.09
Min Buri	3/35	8.6	1.80–23.08
Lat Krabang	2/32	6.3	0.76–20.81
Lak Si	2/34	5.9	0.72–19.68
Bangkok Noi	1/18	5.6	0.14–27.29
Phra Nakhon	1/25	4.0	0.10–20.35
Bang Khun Thian	1/26	3.9	0.10–19.64
Chatuchak	1/30	3.3	0.08–17.22
Sai Mai	1/33	3.0	0.08–15.76
Bang Khen	1/71	1.4	0.04–7.57
Don Mueang	0/24	0.0	0.0–14.25
Lat Phrao	0/42	0.0	0.0–8.41
Nong Chok	0/28	0.0	0.0–12.34
Khlong Sam Wa	0/28	0.0	0.0–12.34
Saphan Sung	0/10	0.0	0.0–30.85
Dusit	0/24	0.0	0.0–14.25
Ratchathewi	0/10	0.0	0.0–30.85
Samphanthawong	0/23	0.0	0.0–14.82
Bang Sue	0/10	0.0	0.0–30.85
Taling Chan	0/19	0.0	0.0–17.65
Bang Kho Laem	0/23	0.0	0.0–14.82
Chom Thong	0/18	0.0	0.0–18.53
Phasi Charoen	0/27	0.0	0.0–12.77
Thon Buri	0/16	0.0	0.0–20.59
Yan Nawa	0/19	0.0	0.0–17.65
Pom Prap Sattru Phai	0/12	0.0	0.0–26.47
Total	16/652	2.5	1.41–3.95

### 3.3 Association with host-related and management-related factors

Male cats showed a slightly higher *T. gondii* DNA prevalence than female cats (2.9% vs 2.1%), although this difference was not statistically significant (χ^2^ = 0.46, *p* = 0.50). Similarly, cats older than 3 years exhibited a higher prevalence (3.1%) compared to cats ≤3 years of age (2.2%), but this association was also not significant (χ^2^ = 0.49, *p* = 0.48) (Table 2).

**Table 2:** Risk factor association with *Toxoplasma gondii* DNA detection in stray cats surrounding Bangkok, Thailand.

Factors	Total (%)	PCR-positive (%)	PCR-negative	Statistical parameters
Genders				χ^2^ = 0.46, df = 1, *p* = 0.50, OR = 1.41, 95% CI = 0.52–3.80
Male	272 (41.7%)	8 (2.9%)	264	
Female	380 (58.3%)	8 (2.1%)	372	
Age group				χ^2^ = 0.49, df = 1, *p* = 0.48, OR = 0.69, 95% CI = 0.25–1.94
≤3 years	459 (70.4%)	10 (2.2%)	449	
>3 years	193 (29.6%)	6 (3.1%)	187	
Cat roaming status				χ^2^ = 0.02, df = 1, *p* = 0.88, OR = 0.89, 95% CI = 0.20–4.01
Free-roaming	578 (88.7%)	14 (2.4%)	564	
Confined	74 (11.3%)	2 (2.7%)	72	
Frequency of feces removal/cleaning				χ^2^ = 0.38, df = 2, *p* = 0.83
Everyday	277 (42.5%)	8 (2.9%)	269	
1–3 times/week	183 (28.1%)	4 (2.2%)	179	
Never	192 (29.4%)	4 (2.1%)	188	
Feeding raw food				χ^2^ = 1.21, df = 1, *p* = 0.27, OR = 2.02, 95% CI = 0.56–7.30
Yes	68 (10.4%)	3 (4.4%)	65	
No	584 (89.6%)	13 (2.2%)	571	
Veterinary care				χ^2^ = 0.18, df = 1, *p* = 0.67, OR = 1.28, 95% CI = 0.41–4.01
Yes	458 (70.2%)	12 (2.6%)	446	
No	194 (29.8%)	4 (2.1%)	190	
Deworming status				χ^2^ = 0.09, df = 1, *p* = 0.76, OR = 1.19, 95% CI = 0.38–3.75
Yes	143 (21.9%)	4 (2.8%)	139	
No	509 (78.1%)	12 (2.4%)	497	
Total	652	16 (2.5%)	636	

Note: OR = odds ratio; CI = confidence interval; df = degrees of freedom.

No statistically significant associations were identified between PCR positivity and assessed management-related factors, including roaming status, frequency of feces removal, feeding of raw food, veterinary care, or deworming status (*p* > 0.05 for all comparisons). Odds ratios did not indicate strong effects for any of the evaluated variables (Table 2).

### 3.4 Molecular confirmation

Among the PCR-positive samples, one representative amplicon targeting the 529 bp repetitive element was successfully sequenced. BLAST analysis confirmed the sequence as *T. gondii*, showing 100% identity with a reference sequence deposited in GenBank (accession number LC547467.1). The sequence generated in this study was submitted to GenBank under accession number PV472053, confirming the specificity of the PCR assay.

## 4 Discussion

Serological surveys of *T. gondii* infection in cats have been widely conducted worldwide, with reported prevalence ranging from very low to nearly 100%, depending on geographic location, cat population, age structure, diagnostic method, and environmental conditions (Dubey et al., 2020). While seroprevalence reflects cumulative exposure to the parasite, it does not provide information on active oocyst shedding or current environmental contamination. Because felids are the only definitive hosts capable of shedding *T. gondii* oocysts, direct examination of fecal samples remains essential for assessing the immediate zoonotic risk posed by cat populations.

In the present study, molecular detection of *T. gondii* DNA in fecal samples from stray cats residing in temple communities across Bangkok revealed a low overall prevalence of parasite DNA. However, PCR-positive samples were detected in more than one-third of the surveyed districts, suggesting that the presence of *T. gondii* is not limited to isolated locations. Additionally, several districts showed zero detected prevalence; however, the corresponding confidence intervals indicate that infection cannot be excluded, highlighting the potential for undetected environmental contamination. However, the detection of *T. gondii* DNA in fecal samples does not necessarily indicate active oocyst shedding, as DNA may originate from recently ingested infected prey (Poulle et al., 2016). The detection of *T. gondii* DNA across multiple districts supports the notion that temple environments may act as potential focal points for environmental contamination, particularly given that oocysts shed by cats can sporulate and remain infective in the environment for prolonged periods (Al-Malki, 2021; Shapiro et al., 2019; Elmore et al., 2010; Robert-Gangneux and Dardé, 2012).

The observed prevalence in this study is lower than that reported in some molecular studies from northern Thailand, where flotation-based oocyst concentration prior to PCR was applied and resulted in substantially higher prevalence (Suwancharoen et al., 2022). The use of fecal swabs in this work may have limited the amount of sample material available for DNA extraction, potentially reducing analytical sensitivity. In addition, no mechanical disruption step was included during DNA extraction. Given the highly resistant nature of *T. gondii* oocyst walls, this may have limited the efficiency of oocyst lysis and DNA recovery. Therefore, the detected DNA may not necessarily originate from intact oocysts. Nevertheless, the detection of *T. gondii* DNA in fecal samples suggests the presence of the parasite and highlights the potential for environmental contamination in areas where humans and animals may be exposed. In contrast, our findings are comparable to those reported from southern Thailand using PCR targeting the 529 bp repetitive element without prior oocyst concentration (Chemoh et al., 2016). Differences in sample processing, molecular targets, and study design likely contribute to this variability. The 529 bp repetitive element used in the present study is a highly sensitive marker due to its high copy number in the *T. gondii* genome (Homan et al., 2000; Reischl et al., 2003). However, the use of parasite DNA as a positive control, although standard practice in molecular assays, may introduce a theoretical risk of contamination. Strict precautions were implemented to minimize this risk, including the use of separate work areas and negative controls. Nevertheless, this possibility cannot be completely excluded.

Only one of the PCR-positive samples yielded a sequence of sufficient quality for species confirmation. Sequence analysis showed 100% identity with reference *T. gondii* strains in GenBank. The limited sequencing success likely reflects low parasite DNA concentrations and the presence of PCR inhibitors commonly found in fecal material, which represent well-recognized limitations of parasite DNA detection in fecal-based molecular studies (Davis et al., 2018). In addition, the use of single-round PCR in fecal samples may have limited detection sensitivity and increased the possibility of nonspecific amplification due to the complexity of the sample matrix. Therefore, the remaining PCR-positive samples should be interpreted in light of the methodological limitations, particularly in the absence of consistent sequencing confirmation, as nonspecific amplification cannot be excluded. Future studies may benefit from optimized DNA extraction protocols, including mechanical disruption steps and oocyst concentration methods, as well as the use of nested or real-time PCR approaches.

Analysis of host-related factors revealed slightly higher prevalence in male cats and in cats older than three years, although these differences were not statistically significant. Previous serological studies have reported higher prevalence in male cats, often attributed to wider roaming ranges and increased exposure opportunities (Hall et al., 2016; Majid et al., 2021; Kengradomkij et al., 2018). Age-related increases in seroprevalence have also been consistently documented (Dubey et al., 2020). In the context of oocyst shedding or detection of parasite DNA from stool, however, such associations are inherently difficult to detect, as cats shed oocysts only once for a short period following primary infection (Dubey, 1995; Dubey, 2022; Lilly and Wortham, 2013). Additionally, accurate age estimation in stray cat populations is challenging and may have contributed to misclassification.

No significant associations were observed between *T. gondii* DNA detection and management-related factors, including roaming status, feeding practices, veterinary care, deworming status, or frequency of feces removal. This finding is consistent with previous reports indicating that no statistically significant relationships between the presence of *T. gondii* DNA and the variables obtained from the questionnaire such as kind of food, water source and deworming (Zamora-Vélez et al., 2020). Given the transient nature of shedding (Dubey, 1995; Dubey, 2022; Lilly and Wortham, 2013), environmental exposure dynamics and prey availability are likely more important determinants than the individual-level factors assessed in this study. However, given the limited number of sequence-confirmed samples and the uncertainty regarding the origin of the detected DNA, the prevalence estimates and statistical analyses should be interpreted cautiously, and the statistical power to detect meaningful associations may be limited.

Importantly, the detection of *T. gondii* DNA in fecal samples from stray cats complements earlier environmental studies reporting the presence of coccidian oocysts in soil from temple grounds across Bangkok (Pinyopanuwat et al., 2018). Together, these findings suggest that temple environments may support sustained environmental contamination through repeated introduction of the parasite into environment by different cats over time, rather than prolonged shedding by individual animals. Such contamination has potential public health relevance, particularly in settings where people regularly access temple grounds and where food and water sources may be exposed to contaminated soil.

Consistent with the transient nature of oocyst shedding during primary infection, parasite DNA was detected at a low prevalence in fecal samples. However, the broad spatial distribution of positive cases underscores the need for continued surveillance of both cat populations and contaminated environments in urban settings. Integrating molecular detection in cats with environmental sampling and human serological data would provide a more comprehensive understanding of *T. gondii* transmission dynamics. Such integrated approaches align with One Health principles and may help inform targeted interventions aimed at reducing environmental exposure to *T. gondii* in densely populated urban communities.

## 5 Conclusion

This study provides molecular evidence of *T. gondii* DNA detection in stool from stray cats residing in temple communities across Bangkok. Although parasite DNA occurred at low prevalence, positive cases were identified across a wide geographic range, suggesting widespread environmental circulation of the parasite in urban settings.

Given the short duration of oocyst shedding in cats and the absence of reliable clinical indicators, molecular detection in fecal samples represents a valuable approach for assessing potential environmental contamination. However, PCR-based detection does not confirm active oocyst shedding and should be interpreted with caution. The presence of *T. gondii* DNA in multiple districts underscores the potential for human and animal exposure, particularly in communal temple environments where contact with contaminated soil, water, or surfaces may occur.

These findings highlight the importance of continued surveillance of stray cat populations and contaminated environments as part of integrated One Health strategies. Combining molecular monitoring in cats with environmental sampling and human serological studies will be essential to better characterize transmission dynamics and to inform targeted public health interventions aimed at reducing the risk of toxoplasmosis in urban communities.

## CRediT authorship contribution statement

**Chanya Kengradomkij:** Writing – original draft, Visualization, Methodology, Investigation, Formal analysis, Data curation. **Wissanuwat Chimnoi:** Investigation, Data curation. **Ketsarin Kamyingkird:** Investigation, Data curation. **Adrian B. Hehl:** Writing – review & editing, Supervision, Conceptualization. **Tawin Inpankaew:** Writing – review & editing, Supervision, Resources, Methodology, Funding acquisition, Conceptualization.

## Declaration of competing interest

The authors declare that they have no known competing financial interests or personal relationships that could have appeared to influence the work reported in this paper.
